# Five-Year-olds' Acoustic Realization of Mandarin Tone Sandhi and Lexical Tones in Context Are Not Yet Fully Adult-Like

**DOI:** 10.3389/fpsyg.2018.00817

**Published:** 2018-05-28

**Authors:** Nan Xu Rattanasone, Ping Tang, Ivan Yuen, Liqun Gao, Katherine Demuth

**Affiliations:** ^1^Department of Linguistics, Macquarie University, Sydney, NSW, Australia; ^2^Center for Language Sciences, Macquarie University, Sydney, NSW, Australia; ^3^ARC Center of Excellence in Cognition and its Disorders, Macquarie University, Sydney, NSW, Australia; ^4^Centre for Speech, Language and the Brain, Beijing Language and Culture University, Beijing, China

**Keywords:** lexical tone acquisition, tone sandhi, mandarin, acoustic analysis, pre-schoolers

## Abstract

Large numbers of children around the world are learning tone languages, but few studies have examined the acoustic properties of children's early tone productions. Even more scarce are acquisition studies on tone sandhi, a tone change phenomenon which alters the surface realization of lexical tones. Two studies using perceptual coding report the emergence of lexical tone and tone sandhi at around 2 years (Li and Thompson, 1977; Hua and Dodd, 2000). However, the only acoustic study available shows that 3-year-olds are not yet adult-like in their lexical tone productions (Wong, 2012). This raises questions about when children's productions become acoustically adult-like and how their tone productions differ from those of adults. These questions were addressed in the current study which compared Mandarin-speaking pre-schoolers' (3–5-year-olds) tone productions to that of adults. A picture naming task was used with disyllabic real words familiar to pre-schoolers. Overall children produced appropriate tone *contours* for all tones, i.e., level for tone 1, rising for tones 2, 3 and full sandhi, falling for tone 4 and half sandhi. However, children's productions were not adult-like for tones 3, 4, and the sandhi forms, in terms of coordinating *pitch range, slope and curvature*, with little evidence of development across ages. These results suggest a protracted process in achieving adult-like acoustic realization of both lexical and sandhi tones.

## Introduction

Despite recent interest in tone languages, little is known about the acquisition of lexical tones compared to segments (i.e., vowels and consonants). This is in spite of the pervasiveness of tone languages—it is estimated that more than half the world's languages are tonal (Yip, 2002). Especially lacking is knowledge about children's early productions of lexical tone, and if and how these differ from adult forms. This is also the case for phonological processes that involve lexical tone change (tone sandhi). For example, Mandarin has tone sandhi processes whereby the surface tone changes depending on tonal context, i.e., from tone 3 to a rising or falling tone. The acquisition of such phonological processes has not attracted much attention in the field of language acquisition. Given the large population of children learning tone languages, understanding how lexical tone and tone sandhi processes are acquired is crucial for providing a comprehensive account of language acquisition above the level of the segment. In this paper, we examine the early production of both lexical and sandhi tones in terms of their acoustic realizations to determine if pre-schoolers' productions are adult-like.

**Figure 1:**
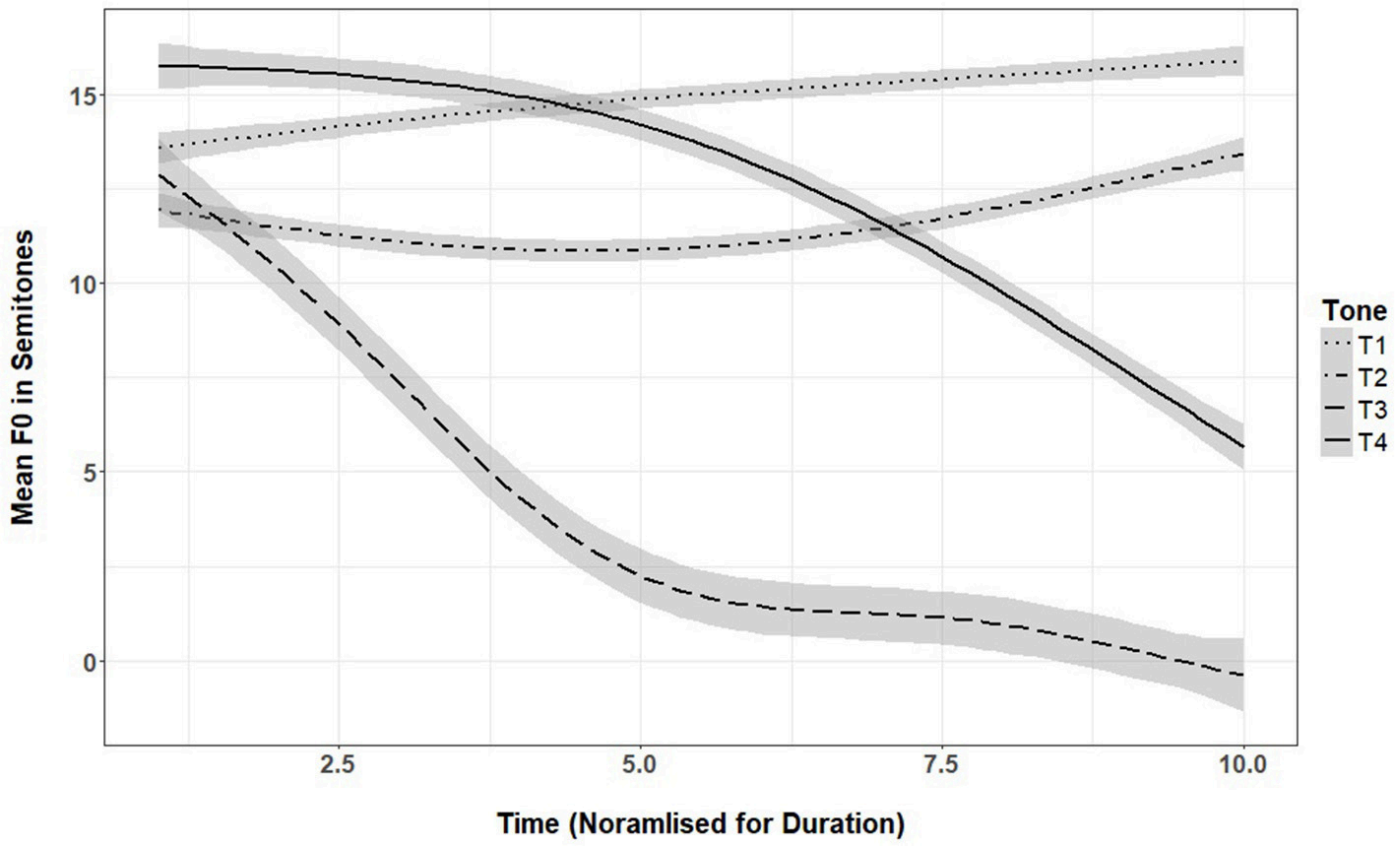
Mean f0 at 10 time points for the four lexical tones produced by 16 female native speakers of Mandarin from Beijing (shaded area is 95% confidence interval).

Languages that have lexical tone manipulate pitch height and pitch contours to change the meanings of words. Whereas in English rising and falling pitch contours on words are typically associated with prosodic information such as intonation and focus, in lexical tone languages these can change the meaning of the word. A well-studied lexical tone language is Mandarin, with the largest population of speakers around the world. Mandarin has a four-tone system with one level and three contour tones; tone 1 has a high level contour (“mā”: mother), tone 2 a rising contour (“má”: hemp), tone 3 a dipping contour (“mǎ”: horse), and tone 4 a falling contour (“mà”: reprimand). See Figure 1 for pitch contours across time on the four lexical tones. While all four lexical tones appear in the productions of Mandarin-speaking children by the 1-word stage of development, confusion between tones 2 and 3 (rising and dipping tones) continues into the 2/3-word stage of development, finally disappearing as longer sentences are produced (Li and Thompson, 1977). Only one study has reported on the acoustic characteristics of lexical tone produced by Mandarin-speaking 3-year-olds in North America (Wong, 2012). Using monosyllabic words, the study showed that 3-year-olds did not yet have adult-like productions in terms of pitch range and slope, especially for tone 3, indicating that young children face challenges in producing complex tonal contours. This is mirrored in perception studies with 3-year-olds showing difficulty with tone identification, especially for the tone 3—having the most complex tone contour (Wong et al., 2005). Another study using perceptual coding of Mandarin-speaking Taiwanese 4- and 5-year-olds' tone productions, showed that older pre-schoolers still had a substantial number of atypical productions, with no changes over development (Wong, 2013). Together these studies suggest that Mandarin-speaking pre-schoolers are still learning to produce tone in an adult-like manner.

These studies point to a protracted acquisition period for Mandarin lexical tones, especially when compared to other tone languages with larger tone inventories. For example, Cantonese is a language with a six-tone system, but children are reported to have acquired all tones by the age of 2 (So and Dodd, 1995). This includes tones with very similar pitch contours, e.g., three level tones (high, mid, and low) and two rising tones (high and mid). The same early acquisition of the full tone inventory is also observed in Thai, a language with 5 tones including three level tones (high, mid, and low), a rising and falling tone, where all tones were present by the 2-word stage (Tuaycharon, 1977). Thus, a larger tone inventory and similarities between tonal contours does not appear to delay tone acquisition. One obvious reason might be that the *perceptual* coding (rather than acoustic analysis) used in these studies overestimated children's abilities. Indeed, during early stages of language acquisition, children can produce acoustic contrasts that may not be detected by the listener (Scobbie et al., 2000). However, another possibility is the role of Mandarin tone sandhi in acquisition. Tone change processes such as tone sandhi, where children must learn to associate multiple surface forms with their underlying forms, might contribute to a protracted acquisition process. However, little is known about the acoustic realizations of children's early productions of tone sandhi, whether it is adult-like and if it is acquired with lexical tones.

**Figure 2:**
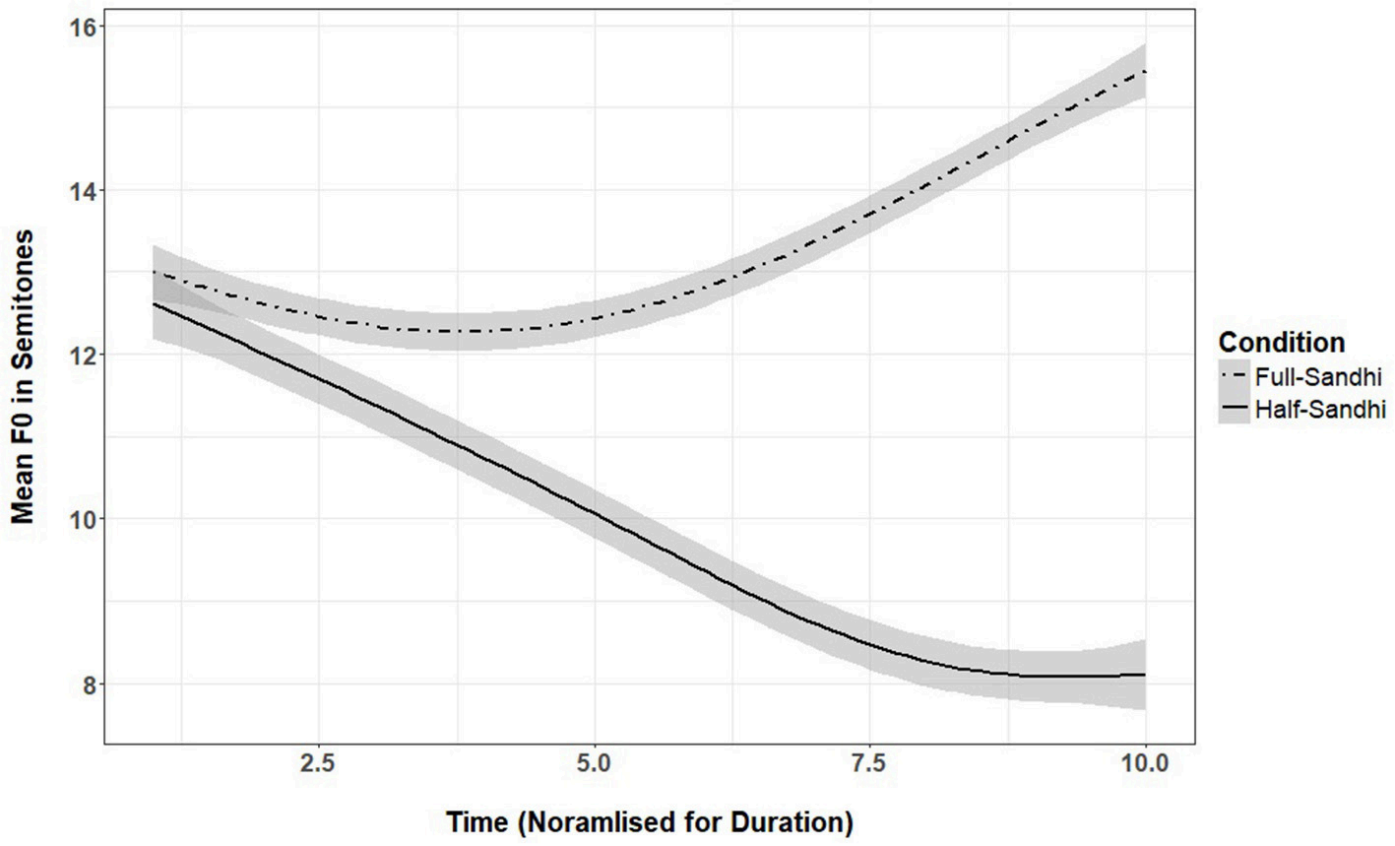
Mean f0 at 10 time points for full- and half-sandhi produced by 16 female native speakers of Mandarin from Beijing (shaded area is 95% confidence interval).

There are two contexts for tone sandhi process in Mandarin. The full sandhi context occurs when two tone 3 syllables occur in succession (tones 3–3), and the first becomes a rising tone. The half sandhi context occurs when tone 3 is followed by any other tone (tones 1, 2, or 4), and is realized with a falling pitch. See Figure 2 for pitch contours plotted over time for the full and half sandhi tones. Therefore, except in the utterance final position, tone 3 is always realized as full or half sandhi in connected speech. Previous studies have reported that tone sandhi *emerges* by the 2/3-word stage of development (around 2 years), when children begin to combine words (Li and Thompson, 1977; Hua and Dodd, 2000). However, it is unclear how sandhi forms are acoustically realized in children's productions. To date, we know of only one study which has reported on the acoustic characteristics of tone sandhi productions by pre-schoolers (Xu Rattanasone et al., 2016). That study reported that 3-year-olds' production of Mandarin tone sandhi on known words had tonal contours that are consistent with the sandhi forms. However, no adult control group was used and so it remains unclear the extent to which 3-year-old's productions are acoustically adult-like. Given previous reports on the protracted acquisition of tone sandhi, it is unlikely that 3-year-olds' productions would be adult-like at this early age. Indeed, in Bantu languages such as Sesotho, where lexical and grammatical tone interact, tone sandhi processes begin to emerge only by 3 years or later, as children learn more about the grammar of the language (Demuth, 1993).

Currently, it is unclear why some studies have reported Mandarin tone acquisition to be a protracted process. This could be related to the presence of a tone sandhi process or difficulty in producing the adult-like forms of both lexical and sandhi tones. One possibility is that children are producing global tonal contours that are consistent with lexical and sandhi tones (level, rising and falling), but are not yet able to make finer acoustic adjustments in an adult-like manner (e.g., pitch range). Indeed, previous studies of 3-year-olds have shown that children are producing global tonal contours that are consistent with lexical and sandhi tone targets (Xu Rattanasone et al., 2016), but these are not yet adult-like in terms pitch range, slope and curvature (Wong, 2012). A recent study reporting on adult ratings of child productions showed that compared to adult productions, children's productions were rated as being less accurate, especially in complex phonetic contexts – disyllables (Wong and Strange, 2017). These complex phonetic contexts include the tone sandhi context, but it is unclear from that study whether children were producing sandhi forms. Therefore, it remains unclear when children might reach adult-like productions on acoustic characteristics of pitch range, slope and curvature for both lexical and sandhi tones in context such as in disyllables.

In this study, we addressed the question of whether pre-schooler's lexical and sandhi tone productions are acoustically adult-like by comparing 3-, 4-, 5-year-olds' productions to adult forms. All participants were monolinguals raised in Beijing. First, we report on lexical tone productions. Based on previous research, we expected that all children might produce global tonal contours that are consistent with the four lexical tones (level, rising, dipping and falling). However, we also predicted that children might not reach adult-like levels on acoustic measures such as pitch range, slope and curvature. We also expected that there might be a developmental effect whereby older 5-year-olds' productions would be more acoustically adult-like than the productions of younger children. Secondly, we report on tone sandhi productions from the same groups of children compared to adults. Based on one previous study (without an adult control group), we expected that children's global tonal contours for tone sandhi productions to be consistent with full and half sandhi forms (rising vs. falling). No study has yet reported on children's productions of pitch range, slope and curvature for tone sandhi, but based on lexical tone, we predicted that children will not be adult-like on these measures. However, 5-year-olds' productions might be more adult-like than younger 3-year-olds.

## Materials and methods

### Participants and design

Participants included 27 3-year-olds with a mean age of 3; 10 (range 3; 5– 3;11; 12 boys and 9 girls), 22 4-year-olds with a mean age of 4; 7 (range 4; 0–4; 11; 7 boys and 16 girls) and 25 5-year-olds with a mean age of 5; 7 (range 5; 0–5; 11; 16 boys and 15 girls). No participants were excluded.

All children were recruited in Beijing from the preschool associated with the Beijing Language and Culture University. The study was conducted in accordance with the ethics protocol approved by Macquarie University's Human Ethics Panel. All child participants received stickers for their participation and the preschool received book donations for all children to use at the center.

A total of 16 adult female controls, with mean age of 24 years (range: 19–35 years) were recruited. All adults are native speakers of Beijing Mandarin and were undertaking graduate or post-graduate training in Sydney. Written consent was provided prior to participation in the study and they were paid $20 for their travel and time.

A within-subjects design was used. All participants were asked to name all lexical tone and tone sandhi items during testing.

### Stimuli

The stimuli included a total of 28 disyllabic words familiar to pre-schoolers (Table 1). To elicit the lexical tones, 12 disyllabic words with tones 1, 2, and 4 on the first syllable and tones 1–4 on the second syllable were chosen. It was not possible to find enough familiar words for pre-schoolers all beginning with tone 1 to avoid tone co-articulation effects, therefore an equal number of words beginning with tones 2 and 4 (rising and falling contours) were also included to elicit a range of tonal contexts. It was also not possible to avoid some words ending in nasal /n/ and /η/ codas, which can have the effect of lowering the pitch of the syllable.

**Table 1:** List of disyllabic stimuli words.

	**Tones**	**Pinyin**	**Meaning**
Practice	T3T3	Xiao-gou	Puppy
	T3T3	Xiao-ma	Pony
Full	T3T3	Lao-shu	Mice
sandhi	T3T3	Lao-hu	Tiger
	T3T3	Xiao-niao	Chick (bird)
	T3T3	Yu-san	Umbrella
Half	T3T1	Xiao-mao	Kitten
sandhi	T3T1	Jian-dao	Scissors
	T3T1	Kao-ya	Peking duck
	T3T1	Bing-gan	Biscuit
	T3T2	Kou-hong	Lipstick
	T3T2	Cai-hong	Rainbow
	T3T2	Cao-mei	Strawberry
	T3T2	Xiao-niu	Calf
	T3T4	Shou-tao	Gloves
	T3T4	Tu-dou	Potatoes
	T3T4	Kong-que	Peacock
	T3T4	Tan-ke	Tanker
Lexical	T1T1	Xi-gua	Watermelon
tone	T1T2	Ying-tao	Cherries
	T1T3	Ban-ma	Zebra
	T1T4	Ji-dan	Egg
	T2T1	Long-xia	Lobster
	T2T2	Liang-xie	Sandals
	T2T3	Ping-guo	Apple
	T2T4	Qin-cai	Celery
	T4T1	Li-zhi	Lychee
	T4T2	Qi-qiu	Balloon
	T4T3	Chi-bang	Wings
	T4T4	Da-xiang	Elephant

To elicit full sandhi, four disyllabic tone 3-3 words were chosen. For half sandhi 12 disyllabic words were chosen, with tone 3 as the first syllable and tones 1, 2, and 4 as the second syllable. This resulted in a total of 16 sandhi items. An additional two practice items in the full sandhi form (a puppy and a pony) were used at the beginning of each session to help train children to performing the task. These training items were not analyzed.

Most syllables had a CV structure, and where possible contained a stop or fricative/affricate onset to facilitate acoustic coding. However, a few contained a lateral or nasal onset, and some contained a nasal coda. Two versions of the test were created, each with a different randomization for the presentation order of words. See Appendix for Table A1 on durations of each tone by syllable.

### Equipment

A total of 34 non-proprietary photographic images representing each of the 32 test and 2 practice items were selected from Google images. The images were presented one at a time using Microsoft PowerPoint 2013 delivered on an Apple iPad 2. The recordings were collected using a Zoom H2 digital voice recorder with lapel mic and the recordings were exported as PCM files.

### Procedure

Testing was conducted in a quiet area in the preschool. Each child was greeted by the native Mandarin-speaking experimenter, the first author. The task was explained as a picture naming game where children named the pictures on an iPad and received stickers for playing the game. Two practice trials were given, and for children who could not provide an answer after three attempts, the experimenter provided the answer, e.g., “puppy.” The child was then asked to repeat the label before moving on to the next item. The children were encouraged to provide answers independently during the practice trials.

All children could perform the elicitation task during the test trials, however, there were two items which most children could not name, i.e., the tones 3-2 word (gloss: rainbow) and tones 2-1 word (gloss: lobster). For these items, the experimenter named the items but the imitations from the children were not analyzed.

The same procedure was used for testing all children as well as the adult control.

### Data analysis

The productions were acoustically coded in Praat (Boersma and Weenink, 2012) by a trained coder who is a native speaker of Mandarin. Two additional native speakers listened to all tokens. No mis-productions on consonant or vowel segments were identified by any of the three listeners so all productions were included and contributed to the final analyses. A total of 60,090 tokens were analyzed, 17,860 from the 3-year-olds, 14,920 from the 4-year-olds, 17,260 from the 5-year-olds, and 11,050 from the adults. The tones were extracted from the vocalic portion of the target syllable (and nasal if present), this was the second syllable for lexical tone words and the first for tone sandhi words. The vocalic portion was identified from the onset to cessation of higher formants. In cases where the second syllable had a nasal onset, anti-resonance and simplification of the waveform was used to identify the onset of the second syllable. F0 points were tracked within the annotated interval, using autocorrelation algorithm in PRAAT, and these f0 points were checked and manually revised to correct for any “doubling” or “halving” errors in pitch tracking. The revised pitch track was then interpolated and smoothed with a bandwidth of 20 Hz. F0 was then extracted in 10 equal steps for each syllable. The raw f0 values were transformed into semitones, with reference frequency of 100 hz, for anlaysis.

## Results

To examine whether children's lexical tone and tone sandhi productions were adult-like, second order polynomial models were conducted for each tone separately (6 models in total: 4 for lexical tones and 2 for sandhi forms). Alpha was set at 0.008 after Bonferroni adjustment was made for multiple comparisons. In all models, children's productions were compared to the adult controls. The first order linear trends compared the steepness of the slopes, and the larger estimates indicated steeper slopes with larger differences between f0 onset and offset, i.e., greater pitch range. The second order quadratic trends compared the areas under the curve, with larger estimates indicating larger areas, i.e., more curvy contours.

Since children have higher pitch than adults, data for each age group was centered around the group means to ensure that only differences in f0 contour is analyzed and not the absolute f0 differences between children and adults. All analyses were conducted in *R* (R Core Team, 2013) using the *lmerTest()* function of the *lme4 package* with *Satterthwaite* adjustments to denominator degrees of freedom (Bates et al., 2015). The model included f0 measured over 10 time points (every 10%) as the dependent variable with Age group (3-, 4-, 5-year-olds, and Adults) as the fixed factor. Each speaker was entered as a random variable with random intercept estimated separately for each age group. The models for lexical tones (1–4) are reported first followed by tone sandhi (full- and half-sandhi). See Tables 2,3 for fixed effects model estimates of lexical tone and tone sandhi as well as R-codes for estimating the maximal model.

**Table 2:** Results for f0 of lexical tone across 10 time points.

**Fixed effects**	**Estimate**	**S.E**.	***df***	***t***	***p***
**TONE 1**					
(Intercept)	0.522	0.540	89.900	0.967	0.336
**Linear**	**2.330**	**0.324**	**87.600**	**7.182**	0.000***
Quadratic	−0.473	0.219	644.000	−2.157	0.031*
Two-way Interactions					
**Linear** × **3-year-olds**	−**1.573**	**0.410**	**88.200**	−**3.837**	0.000***
**Linear** × **4-year-olds**	−**1.716**	**0.427**	**88.400**	−**4.017**	0.000***
Linear × 5-year-olds	−0.787	0.417	89.300	−1.885	0.063
Quadratic × 3-year-olds	0.105	0.278	652.700	0.377	0.706
Quadratic × 4-year-olds	0.248	0.290	656.400	0.857	0.392
Quadratic × 5-year-olds	0.197	0.284	668.400	0.695	0.487
**TONE 2**					
(Intercept)	−2.698	0.538	89.700	−5.012	0.000***
**Linear**	**1.554**	**0.521**	**87.800**	**2.980**	0.004***
**Quadratic**	**2.199**	**0.284**	**694.600**	**7.733**	0.000***
Two-way Interactions					
**Linear** × **3-year-olds**	**1.859**	**0.662**	**90.100**	**2.808**	0.006**
Linear × 4-year-olds	1.064	0.687	88.600	1.549	0.125
Linear × 5-year-olds	1.717	0.669	88.500	2.566	0.012*
Quadratic × 3-year-olds	−0.824	0.367	751.000	−2.247	0.025*
Quadratic × 4-year-olds	−0.755	0.377	717.000	−2.002	0.046*
**Quadratic** × **5-year-olds**	−**1.193**	**0.367**	**714.100**	−**3.251**	0.001**
**TONE 3**					
(Intercept)	−10.264	0.841	89.250	−12.198	0.000***
**Linear**	−**12.784**	**0.927**	**85.450**	−**13.785**	0.000***
**Quadratic**	**4.929**	**0.690**	**84.580**	**7.143**	0.000***
Two-way Interactions					
**Linear** × **3-year-olds**	**7.950**	**1.183**	**89.340**	**6.718**	0.000***
**Linear** × **4-year-olds**	**9.959**	**1.229**	**88.200**	**8.106**	0.000***
**Linear** × **5-year-olds**	**10.251**	**1.190**	**86.270**	**8.611**	0.000***
Quadratic × 3-year-olds	0.285	0.888	91.690	0.321	0.749
Quadratic × 4-year-olds	−0.842	0.920	89.420	−0.915	0.363
Quadratic × 5-year-olds	−2.094	0.888	86.060	−2.360	0.021*
**TONE 4**					
(Intercept)	−2.092	0.504	89.670	−4.149	0.000***
**Linear**	−**10.151**	**0.679**	**89.960**	−**14.947**	0.000***
**Quadratic**	−**3.635**	**0.335**	**154.030**	−**10.850**	0.000***
Two-way Interactions					
**Linear** × **3-year-olds**	**5.876**	**0.859**	**90.660**	**6.842**	0.000***
**Linear** × **4-year-olds**	**6.839**	**0.895**	**90.770**	**7.645**	0.000***
**Linear** × **5-year-olds**	**6.770**	**0.871**	**90.630**	**7.770**	0.000***
**Quadratic** × **3-year-olds**	**3.491**	**0.426**	**159.280**	**8.188**	0.000***
**Quadratic** × **4-year-olds**	**3.724**	**0.445**	**160.080**	**8.376**	0.000***
**Quadratic** × **5-year-olds**	**3.729**	**0.433**	**159.140**	**8.622**	0.000***

*** p < 0.001,

** p < 0.01,

* p < 0.05.

Bold effects are still significant after Bonferoni adjustment (alpha < 0.008). R-code: lmer(f0Centered ~ (Linear+Quadratic)*AgeGroup + (Linear+Quadratic|Participant:AgeGroup)).

**Table 3:** Results for f0 of tone sandhi across 10 time points.

**Fixed Effects**	**Estimate**	**S.E**.	***df***	***t***	***p***
**FULL-SANDHI**					
(Intercept)	−1.083	0.453	89.000	−2.389	0.019*
**Linear**	**2.721**	**0.412**	**79.300**	**6.609**	0.000***
**Quadratic**	**1.963**	**0.220**	**313.500**	**8.928**	<2e-16***
Two-way Interactions					
Linear × 3-year-olds	0.331	0.530	86.000	0.625	0.534
Linear × 4-year-olds	−0.529	0.550	84.400	−0.963	0.338
Linear × 5-year-olds	−0.667	0.531	81.500	−1.256	0.213
**Quadratic** × **3-year-olds**	−**1.238**	**0.297**	**404.500**	−**4.168**	0.000***
**Quadratic** × **4-year-olds**	−**1.245**	**0.304**	**378.600**	−**4.092**	0.000***
**Quadratic** × **5-year-olds**	−**1.166**	**0.288**	**341.600**	−**4.045**	0.000***
**HALF-SANDHI**					
(Intercept)	−4.480	0.574	90.100	−7.800	0.000***
**Linear**	−**5.010**	**0.424**	**91.100**	−**11.822**	0.000***
**Quadratic**	**0.817**	**0.244**	**813.100**	**3.347**	0.001***
Two-way Interactions					
**Linear** × **3-year-olds**	**2.415**	**0.533**	**90.100**	**4.528**	0.000***
**Linear** × **4-year-olds**	**2.734**	**0.555**	**89.900**	**4.925**	0.000***
**Linear** × **5-year-olds**	**2.824**	**0.542**	**90.300**	**5.214**	0.000***
Quadratic × 3-year-olds	0.171	0.306	787.900	0.558	0.577
Quadratic × 4-year-olds	0.137	0.318	784.700	0.430	0.667
Quadratic × 5-year-olds	0.040	0.311	794.800	0.128	0.898

*** p < 0.001,

** p < 0.01,

* p < 0.05.

Bold effects are still significant after Bonferoni adjustment (alpha < 0.008). R-code: lmer(f0Centered ~ (Linear+Quadratic)*AgeGroup + (Linear+Quadratic|Participant:AgeGroup)).

### Lexical tones

**Figure 3:**
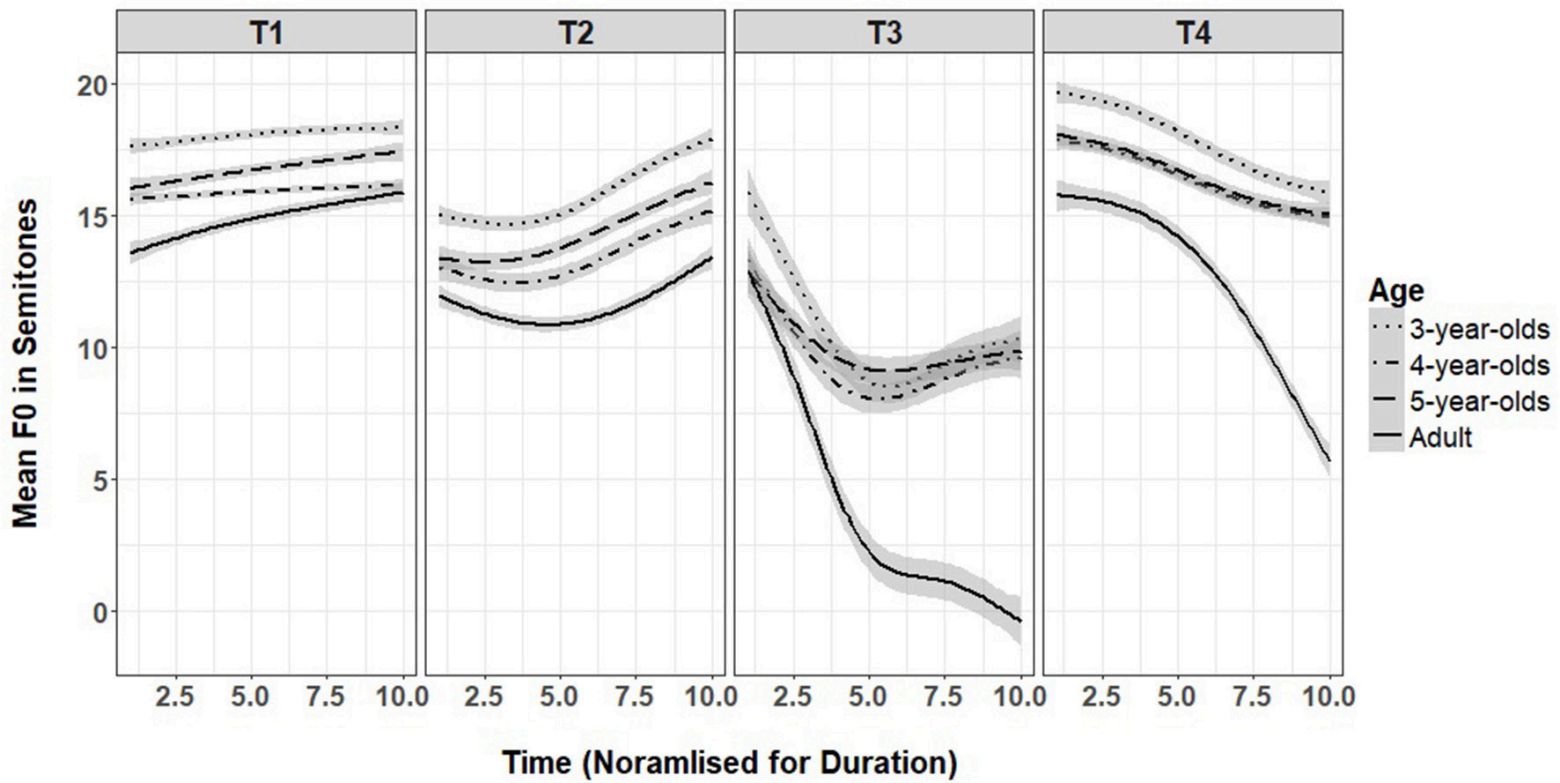
Mean f0 at 10 time points for the four lexical tones by three child ages and for adults (shaded area is 95% confidence interval).

We predicted that children would produce global tonal contours consistent with level, rising, dipping and falling tones but will not be adult-like in producing pitch range, slope and curvature. We also predicted that older 5-year-olds' productions might be more adult-like than younger 3-year-olds. The results for lexical tone are shown in Table 2 and Figure 3. After Bonferroni adjustments for multiple models (6, 4 lexical tones and 2 sandhi tones), alpha was set at 0.008.

For tone 1, there was a significant linear trend and its interaction with age. The significant linear trend in the absence of a significant quadratic trend suggests that tone 1 productions from children and adults had a level contour, consistent with the contour expected for tone 1. The linear interaction with 3- and 4-year-olds, with significant negative estimates compared to adults, suggest that children's tone 1 productions had a flatter slope than adults.

The results for lexical tone 2 showed significant linear and quadratic trends suggesting that both children and adults produced a curved rising f0 contour. There were significant interactions with age for both the linear and quadratic trends. The positive effect on the linear term for 3-year-olds suggest that they produced f0 contours with steeper slopes than adults and therefore, a larger f0 range. The significant negative effect on the quadratic term for 5-year-olds suggest they produced a flatter f0 curve than adults. No other significant interactions were found.

The results for lexical tone 3 showed significant linear and quadratic trends suggesting that both children and adults produced a curved falling f0 contour. Since tone 3 has a negative going contour, the positive effect on the linear term for all three child ages suggest that children had a flatter f0 slope compared to adults, and produced tone 3 with a smaller f0 range. There were no significant interactions with the quadratic trend which suggests that the curviness of the f0 contours in the child and adult productions did not differ.

The results for tone 4 showed significant main effects for both linear and quadratic trends and interactions with age for all three age groups. The linear and quadratic trends suggest that both child and adults produced a curved falling f0 contours. The positive effect of all child groups on the linear and quadratic terms suggest that children produced flatter f0 curves and slope compared to adults, with reduced f0 range.

Overall, the results on lexical tones suggest that children were adult-like for producing global tonal contours consistent with a level contour for tone 1, rising for tone 2, dipping for tone 3 and falling for tone 4. They were also adult-like on f0 range, slope and curvature for tone 1, and mostly adult-like for tone 2. However, all children produced tone 3 with flatter slope and reduced f0 range compared to adults. Children's production of tone 4 differed the most from that of adults, with children producing both reduced f0 range and flatter f0 curves. The results did not show any consistent developmental changes across age, suggesting that older 5-year-olds were not more adult-like in their productions than younger 3-year-olds.

### Tone sandhi

**Figure 4:**
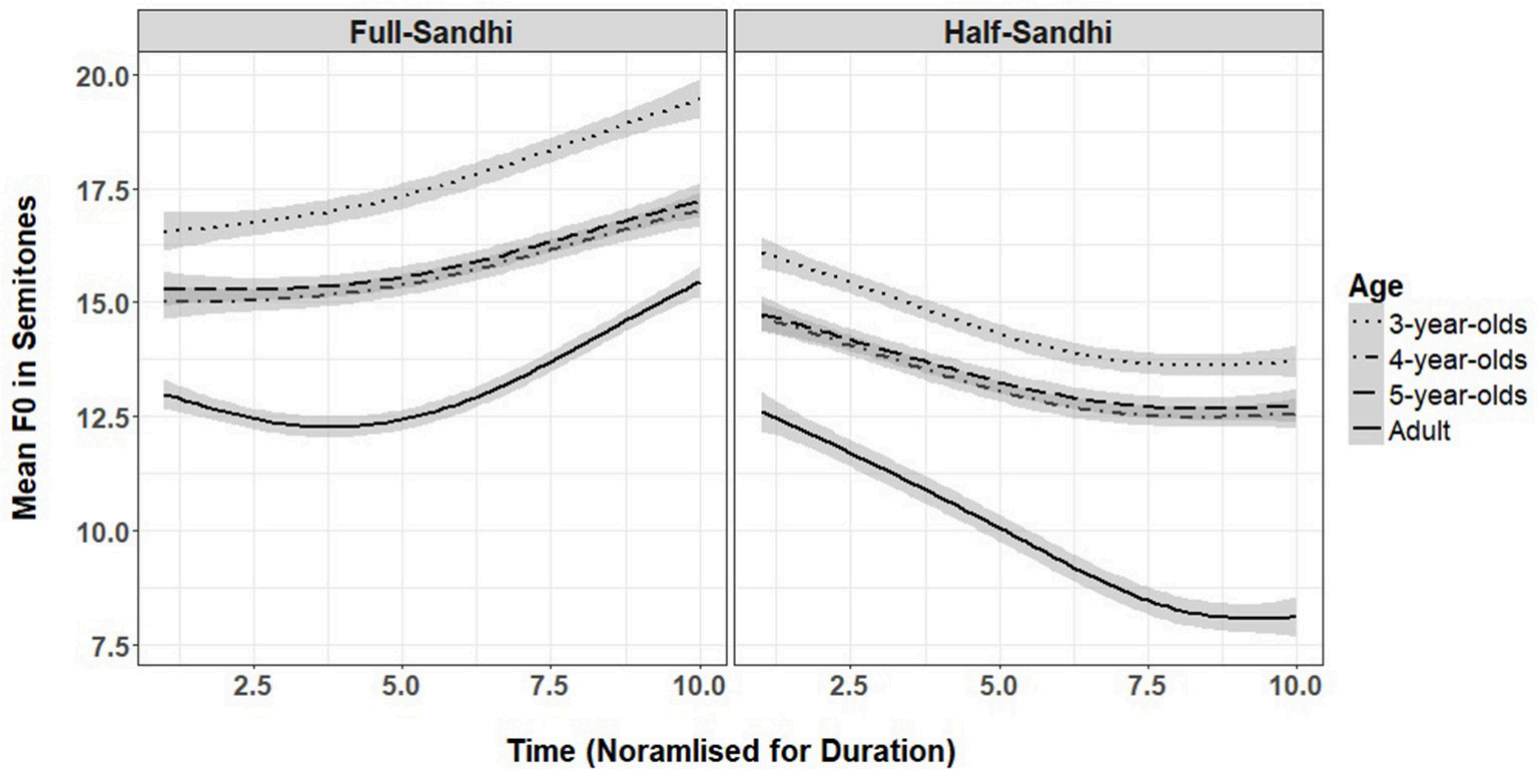
Mean f0 at 10 time points for full- and half-sandhi by three child ages and for adults (shaded area is 95% confidence interval).

We predicted that children might produce the correct global tonal contours that are consistent with full and half sandhi (rising and falling) but would not be adult-like in producing pitch range, slope and curvature. However, children's productions might be more adult-like for older 5-year-olds than younger 3-year-olds. The results for the sandhi forms are shown in Table 3 and Figure 4.

For full sandhi, there was a significant main effect of linear and quadratic trends and an interaction with age for the quadratic trend. The linear and quadratic trends suggest that both children and adults produced curved rising f0 contours. The negative effects of all child groups on the quadratic term suggest that children produced full sandhi with flatter f0 contours than adults.

The results for half sandhi showed significant main effects of linear and quadratic trends and a significant interaction with age for the linear trend. The linear and quadratic trends suggest that both children and adults produced curved falling f0 contours. The positive effects of all child groups on the linear term suggest that children produced half sandhi with flatter f0 slopes and reduced f0 range compared to adults. These results suggest that children are not yet adult-like in their tone sandhi productions for f0 range, slope and contour, even for the oldest age group.

## Discussion

The aim of this study was to examine the acoustic realizations of lexical and sandhi tones in the productions of pre-schoolers (3-, 4-, and 5-year-olds) to determine if and when they become adult-like. First, all global contours on the children's lexical tone productions were consistent with the productions by adults: a level contour for tone 1, a curved rising contour for tone 2, a curved downward dipping contour for tone 3, and a falling contour for tone 4.

However, in terms of pitch range, slope and curvature, the acoustic analysis of lexical tones suggest that children were not achieving adult-like productions across all tones. While child and adult productions of tones 1 and 2 were the least different, tones 3 and 4 showed much more difference between child and adult productions. For tone 1, 3-, and 4-year-olds produced pitch contours with smaller pitch range and flatter pitch contour compared to adults, but were adult-like by 5 years. Tone 2 also showed few differences between child and adult productions with 3-year-olds producing a larger pitch range and slope compared to adults, and 5-year-olds producing a contour that is less curvy compared to adults. No other group differences were found. Therefore, for tone 2, despite having a curved contour, most pre-schoolers produced it in an adult-like manner consistent with a rising tone.

Children's productions of tones 3 and 4 differed the most from adult productions in terms of pitch range, slope and curvature. For tone 3, children across all three age groups had a reduced pitch range and slope compared to adults. However, children did not show any challenges in producing curved contours for the complex tone 3; in fact, the degree of curvature did not differ from adult productions. For tone 4, all children's productions were reduced in pitch range and slope, as well as having a flatter contour with less curvature compared to adult productions. These results suggest that for the two lexical tones with a falling contour, tones 3 and 4, pre-schoolers are still struggling to coordinate pitch range, slope and curvature, even at the age of 5.

The second aim of this study was to examine the acoustic realizations of tone sandhi in children's productions. The analyses for both full and half sandhi suggest that children produced global tonal contours that are consistent with full and half sandhi tones (rising and falling). However, children are not yet adult-like on pitch range, slope and curvature. Compared to adults, children produced full sandhi contours with a flatter rising curve and half sandhi contours with a smaller falling pitch range and slope, again showing challenges in producing adult-like forms.

The results from both lexical tone and tone sandhi suggest that children are still fine-tuning their control and coordination of pitch range and slope with curvature, especially for tones 3, 4, and the sandhi forms. This provides support for Wong (2012, 2013) and suggests that reaching adult-like tone realization on specific acoustic measures is a protracted process. However, our study also found that even 3-year-olds could produce the overall tonal contours consistent with level, rising, dipping and falling tones, important for maintaining tone category distinctions. This may help explain why studies using perceptual coding have reported earlier acquisition of lexical tones compared to studies using acoustic measures; the former may have captured children's ability to produce global tonal contours that are consistent with the different tone categories (Hua and Dodd, 2000), whereas the latter identified the implementation of specified acoustic measures (pitch range, slope and curvature) that are not yet adult-like (Wong, 2012). Together with our study, these results suggest the gradual acquisition of tone realization, with children producing global contours first, and later fine-tuning of pitch range, slope and curvature. Studies with older children, and on tonal coarticulation in a range of tone contexts will be needed to determine when this fine-tuning reaches adult-like acoustic values.

Similarly, for tone sandhi, children are producing rising and falling contours consistent with the two tone sandhi forms, but still fine-tuning pitch range, slope and curvature. However, our study used only real words and avoided low frequency words which pre-schoolers might not know. It is therefore possible that the sandhi forms examined here were lexicalized as tone 2 for full sandhi and a phonetic variant of tone 3 for half sandhi without children fully understanding how and where sandhi processes apply. Therefore, future studies are needed to examine children's ability to apply tone sandhi processes to novel words, examining their ability to generalize their knowledge about these phonological processes to word learning.

Our study did not find any developmental effects for either lexical tones or tone sandhi forms. Therefore, some caution must be taken when interpreting the results on differences observed across the age groups. For example, the results showed that for T2, 3-year-olds produced a more rising contour and 5-year-olds produced a less curvy contour, but there were no overall developmental effects. This must be interpreted with the general result showing that children's productions are not adult-like for any contour tones (i.e., tones 3 and 4, and full and half sandhi). The differences across age groups might therefore be part of children's general early difficulty in coordinating pitch range, slope and curvature to achieve adult-like productions, with the exception of the level T1 where children had achieved adult-like production by 5 years. However, the question of developmental changes in tone productions would be better answered in future longitudinal studies that track children as they develop mastery over tone production.

Finally, the lack of developmental changes for tone sandhi might be related to the use of known words in this study. It is possible that children might show developmental effects in their ability to apply tone sandhi processes when learning new words using novel items. Our results also raise questions about if and how non-adult-like productions may affect children's tone comprehension abilities. It is possible that children are less sensitive to changes in pitch range, slope and curvature but can track overall tonal contours. It is also possible that other acoustic cues are being favored by children, i.e., duration and turning point for the contour tones. Addressing these questions in future research will provide a comprehensive understanding of tone acquisition and the link between production and perception.

## Conclusion

Mandarin-speaking children produced adult-like global tone contours for lexical tone and tone sandhi were consistent with the level (tone 1), rising (tone 2 and full sandhi), dipping (tone 3), and falling (tone 4 and half sandhi) tone categories, showing that 3–5-year-olds have good knowledge about lexicalized forms of lexical tone and tone sandhi. However, pre-schoolers are still fine-tuning their control over coordinating pitch range, slope and curvature, especially for contour tones 2, 3, and 4, and the sandhi forms. Achieving adult-like acoustic realizations of lexical tone and tone sandhi is a protracted process, probably fully attained after the age of 5.

## Author contributions

NX project leader developed research question, designed experiments, collected data, performed data analysis and write up of drafts for this paper in collaboration with the coauthors. PT assisted in coding data, data analysis and interpretation, and contribution to drafts of the manuscripts. IY contributed to the design and implementation of the stimuli. Assisted in training coders for acoustic coding of the data. Contributed to drafts of the manuscripts. LG assisted in recruitment and data collection. Contributed to drafts of the manuscripts. KD contributed to shaping the research question, stimuli and research design, and to drafts of the manuscript.

## Conflict of interest statement

The authors declare that the research was conducted in the absence of any commercial or financial relationships that could be construed as a potential conflict of interest.

## Appendix

**Table A1:** Mean and SD of durations in millisecond for all tones in the 1st and 2nd syllable positions.

		**Mean duration (ms)**		**SD of duration (ms)**	
**Age**	**Tone**	**1st syllable**	**2nd syllable**	**1st syllable**	**2nd syllable**
3-year-olds	T1	169	278	73	79
	T2	233	309	70	91
	T3	163	322	65	120
	T4	125	254	46	97
4-year-olds	T1	177	293	71	70
	T2	242	327	54	94
	T3	174	315	64	125
	T4	135	263	57	97
5-year-olds	T1	198	293	88	96
	T2	245	341	70	113
	T3	180	320	74	136
	T4	141	287	51	122
Adults	T1	162	283	73	78
	T2	112	262	53	90
	T3	182	235	74	92
	T4	167	210	62	66

*Acoustic analyses were conducted on the 1st Syllable for Tone Sandhi and 2nd syllable for Lexical Tones*.

## References

[B1] Bates D. Mächler M. Bolker B. Walker S. (2015). Fitting linear mixed-effects models using lme4. J. Stat. Softw. 67, 1–48. 10.18637/jss.v067.i01

[B2] Boersma P. Weenink D. (2012). Praat: Doing Phonetics by Computer [Computer program] (Version 5.3.23). Available online at: http://www.praat.org/

[B3] Demuth K. (1993). Issues in the acquisition of the Sesotho tonal system. J. Child Lang. 20, 275–301. 10.1017/S030500090000828X 8376470

[B4] Hua Z. Dodd B. (2000). The phonological acquisition of Putonghua (Modern Standard Chinese). J. Child Lang. 27, 3–42. 10.1017/S030500099900402X 10740966

[B5] Li C. N. Thompson S. A. (1977). The acquisition of tone in Mandarin-speaking children. J. Child Lang. 4, 185–199. 10.1017/S.0305000900001598

[B6] R Core Team (2013). R: A Language and Environment for Statistical Computing. [Methodology Reference]. Available online at: http://www.eea.europa.eu/data-and-maps/indicators/oxygen-consuming-substances-in-rivers/r-development-core-team-2006 (Accessed August 23, 2016)

[B7] Scobbie J. Gibbon F. Hardcastle W. Fletcher P. (2000). Covert contrast as a stage in the acquisition of phonetics and phonology, in Papers in Laboratory Phonology V: Acquisition and the Lexicon, eds Broe M. Pierrehumbert J. (Cambridge: Cambridge University Press), 194–207.

[B8] So L. K. H. Dodd B. J. (1995). The acquisition of phonology by Cantonese-speaking children. J. Child Lang. 22, 473–495. 10.1017/S0305000900009922 8789511

[B9] Tuaycharon P. (1977). The Phonetic and Phonological Development of a Thai Baby: From Early Communicative Interaction to Speech. London, UK: University of London.

[B10] Wong P. (2012). Acoustic characteristics of three-year-olds' correct and incorrect monosyllabic Mandarin lexical tone productions. J. Phon. 40, 141–151. 10.1016/j.wocn.2011.10.005

[B11] Wong P. (2013). Perceptual evidence for protracted development in monosyllabic Mandarin lexical tone production in preschool children in Taiwan. J. Acoust. Soc. Am. 133, 434–443. 10.1121/1.4768883 23297915

[B12] Wong P. Schwartz R. G. Jenkins J. J. (2005). Perception and production of lexical tones by 3-year-old, Mandarin-speaking children. J. Speech Lang. Hear. Res. 48, 1065–1079. 10.1044/1092-4388(2005/074) 16411796

[B13] Wong P. Strange W. (2017). Phonetic complexity affects children's Mandarin tone production accuracy in disyllabic words: a perceptual study. PLoS ONE 12:e0182337. 10.1371/journal.pone.0182337 28806417 PMC5555563

[B14] Xu Rattanasone N. *Tang P. Yuen I. Gao L. Demuth K. (2016). 3-year-olds produce pitch contours consistent with mandarin tone 3 Sandhi, in Proceedings of the 16th Australasian International Conference on Speech Science and Technology, eds Carignan C. Tyler M. D. (Sydney: Australasian Speech Science and Technology Assoc), 5–8.

[B15] Yip M. (2002). Tone. Cambridge: Cambridge University Press.

